# A genome-wide association study to identify genetic markers associated with endometrial cancer grade

**DOI:** 10.1186/1897-4287-10-S2-A47

**Published:** 2012-04-12

**Authors:** T O’Mara, D Duffy, DJ Thompson, S Ahmed, K Ferguson, CS Healey, G Montgomery, M Shah, J Morrison, PP Pharoah, AM Dunning, PM Webb, DF Easton, AB Spurdle

**Affiliations:** 1 Hormone Dependent Cancer Program, Institute of Health and Biomedical Innovation, Brisbane, Australia; 2 Division of Genetics and Population Health, Queensland Institute of Medical Research, Brisbane, Australia; 3 Department of Public Health and Primary Care, University of Cambridge, Strangeways Research Laboratory, Cambridge, UK; 4 Department of Oncology, University of Cambridge, Strangeways Research Laboratory, Cambridge, UK

## 

Endometrial cancer is the most commonly diagnosed gynaecological cancer. Although endometrioid endometrial cancer (80% of cases) generally carries a good prognosis, some patients with this tumour subtype relapse within two years. Identifying genetic variants associated with prognosis could inform clinical decision-making for management at diagnosis, and inform development of chemotherapeutic agents targeting aggressive disease. Genome-wide association studies (GWAS) have been successful in identifying common genetic variation involved in cancer susceptibility. Presently there are limited published studies using GWAS data to identify single nucleotide polymorphisms (SNPs) associated with tumour prognostic indicators, such as grade. We used case data from an endometrial cancer case-control GWAS to assess association of SNPs with endometrial cancer grade. Genome-wide genotyping of 1285 Australian and British women with endometrioid endometrial cancer and reporting Caucasian ethnicity was performed using the Illumina 610K BeadChip. After applying quality control measures, data on 583,366 SNPs for 1220 cases with grade information were used in the analysis. PLINK software was used to assess SNP association with grade (1, 2 or 3), adjusting for study group (Australian or British). Fifty-seven SNPs were found to be significant at <10^–4^. Two variants with evidence of association with higher endometrial cancer grade (p-trend<10^–6^) have been selected for validation in independent sample sets. These SNPs are located in or near genes not previously reported to be involved in cancer aetiology or prognosis and, if confirmed, would represent novel gene targets. Neither of these SNPs fall into the top 1500 SNPs prioritised for validation of association with risk. Results to date suggest that genetic alleles associated with prognostic features, such as cancer grade, may be distinct from those associated with predisposition. GWAS analysis of tumour prognostic features is thus likely to improve understanding of biological pathways influencing outcome for endometrial cancer patients.

